# Flushing‐Assisted Saline Immersion Endoscopic Submucosal Dissection in the Rectum: A Video‐Based Feasibility Series

**DOI:** 10.1002/deo2.70447

**Published:** 2026-09-30

**Authors:** Thomas Matthews, Jonathan Landy, Hiromitsu Ban, Takashi Toyonaga, Noriko Suzuki

**Affiliations:** ^1^ Wolfson Unit for Endoscopy, St Mark's The National Bowel Hospital London UK; ^2^ Royal College of Physicians of Ireland Dublin Ireland; ^3^ Department of Gastroenterology Department of Gastroenterology Omi Medical Center Shiga Japan; ^4^ Division of Gastroenterology Kobe University Graduate School of Medicine Hyogo Japan; ^5^ Imperial College London London UK

**Keywords:** endoscopic submucosal dissection, hemostasis, isotonic solutions, rectal neoplasms, rectum, surgical

## Abstract

Flushing endoscopic submucosal dissection (ESD) is a refinement of saline immersion ESD in which energy‐synchronized saline flushing from the knife tip is hypothesized to disperse micro‐bubbles and facilitate more controlled coagulation at the tissue interface. We retrospectively evaluated the technical feasibility of this approach in 10 rectal procedures performed at a tertiary referral center. Complete procedural videos were reviewed to describe treated visible submucosal vessel burden, minor bleeding, episodes of transient loss of visualization, and hemostatic forceps use. En bloc and histologically margin‐negative (R0) resection was achieved in all 10 cases, while curative endoscopic resection was achieved in nine cases (90%). No intraprocedural perforation, delayed bleeding, post‐ESD coagulation syndrome, or unplanned repeat intervention occurred. Median lesion size was 55 mm (interquartile range, 40–69.5), median procedure duration was 102.5 min (100–167.5), and median treated visible vessel count was 21 (11.5–40.75), corresponding to 1.04 vessels/cm^2^ (0.64–1.74). Minor bleeding and transient loss of visualization occurred during procedures but were controlled endoscopically. Hemostatic forceps were required therapeutically for active bleeding from one vessel across the series, and no procedure required abandonment or conversion to surgery. These findings support the technical feasibility of rectal flushing‐assisted saline immersion ESD. The video‐derived hemostatic measures are descriptive and may provide candidate endpoints for future prospective comparative studies; they do not establish comparative efficacy, hemostatic benefit, or safety.

**Trial Registration**: N/A.

## 1 Introduction

### 1.1 Background

Endoscopic submucosal dissection (ESD) is increasingly used for en bloc resection of large superficial colorectal lesions. Bleeding during ESD can interrupt the dissection plane, obscure visualization, require device exchange, and prolong the procedure. Techniques that improve visualization and facilitate controlled hemostasis may therefore be useful, particularly during dissection around visible submucosal vessels.

Saline immersion ESD provides buoyancy and may improve the endoscopic view within the submucosal plane. However, electrosurgical energy application in a fluid environment may be accompanied by micro‐bubble accumulation at the point of energy delivery, potentially precipitating uncontrolled spark discharge. Flushing‐assisted saline immersion ESD, referred to in this manuscript as Flushing ESD, is a refinement in which saline is synchronously flushed directly from the knife tip during electrosurgical energy application (Figure 1 and Video S1). This approach is hypothesized to disperse micro‐bubbles, reduce spark discharge, and facilitate direct coagulation of target tissue.

**FIGURE 1:**
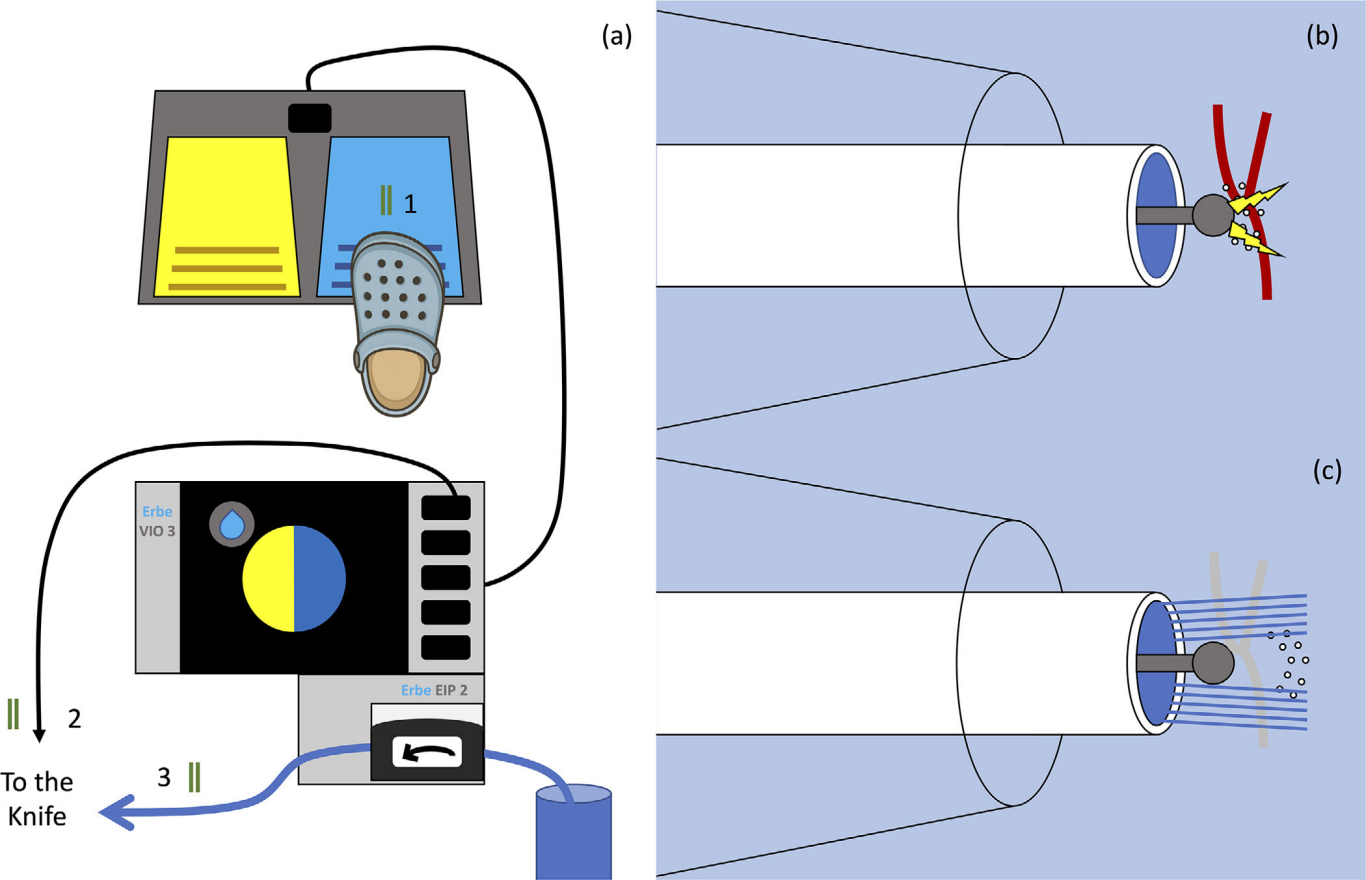
(a) Depression of the blue pedal (1) activates electrosurgical energy delivery at (2) while simultaneously flushing saline through the channel to the knife tip (3). The parallel symbols (||) indicate that these events occur synchronously. (b) Proposed mechanism without saline flushing: micro‐bubbles may accumulate at the tissue interface, potentially promoting electrical spark discharge and local tissue injury. (c) Proposed mechanism with concurrent saline flushing: flushing may disperse micro‐bubbles, reduce spark discharge, and permit more controlled energy delivery to the target vessel. Figure 1 alt text Schematic illustration of flushing‐assisted saline immersion endoscopic submucosal dissection. Panel A shows foot‐pedal activation of electrosurgical energy with simultaneous saline irrigation through the knife channel to the knife tip. Panel B depicts the proposed mechanism without saline flushing, in which micro‐bubble accumulation may promote spark discharge at the tissue interface. Panel C depicts the hypothesized effect of concurrent saline flushing, which may disperse micro‐bubbles, reduce spark discharge, and permit more controlled energy delivery to the target vessel.

The Flushing Forced Method and related principles of synchronized irrigation during electrosurgical treatment under saline immersion have been described previously in technical and case‐based reports [1, 2, 3]. The present study does not describe a new technique; rather, it reports the application of the method across a series of rectal ESD procedures at a European tertiary referral center outside the originating centers, together with a systematic video‐based framework in which bleeding‐related events are normalized to treated visible‐vessel burden and resection area.

### 1.2 Objectives

The aim of this study was to describe the technical feasibility of rectal Flushing ESD. Video‐derived vessel and bleeding metrics were collected to characterize the procedural course and are reported descriptively.

## 2 Procedure or Technique

### 2.1 Study Design, Setting and Participants

We conducted a retrospective descriptive video‐based case series of rectal ESD procedures performed using Flushing ESD. The study was conducted at St Mark's The National Bowel Hospital, London, United Kingdom, a tertiary referral center specializing in complex therapeutic lower gastrointestinal endoscopy.

The rectum was selected a priori as the initial setting for evaluating Flushing ESD because its relatively vascular submucosal plane provided a clinically relevant setting for descriptive assessment of bleeding‐related procedural events. Stable scope position and dependent fluid pooling with appropriate patient positioning also facilitated maintenance of a consistent saline immersion field.

The study was conceived as an exploratory descriptive evaluation of technical feasibility and video‐derived hemostatic metrics.

### 2.2 Participants

Between May 8 and October 30, 2025, the two study endoscopists performed 16 rectal ESD procedures, of which 13 were performed using Flushing ESD. Complete procedural video was available for 10 of the 13 Flushing ESD procedures (76.9%), and all 10 were included in the analysis. The remaining three procedures were excluded because complete procedural video was unavailable. A contemporaneous screening log recording detailed reasons for the absence or incompleteness of individual recordings was not maintained, and these reasons could not be reconstructed reliably.

### 2.3 Technique

Flushing ESD was performed using the previously described Flushing Forced Method [1, 2, 3]. A FlushKnife BTs (Fujifilm Corp., Japan) with integrated irrigation capability was connected to an EIP2 endoscopic irrigation pump (Erbe Corp., Germany), operated at an output setting of 20%, enabling synchronized saline flushing directly from the knife tip during electrosurgical energy application rather than through the endoscopic waterjet channel. The hypothesized effect was to disperse micro‐bubbles at the point of energy delivery, reduce electrical spark discharge, and facilitate more controlled tissue treatment in a saline immersion environment (Figure 1c). Throughout this manuscript, “Flushing ESD” denotes application of the Flushing Forced Method within a saline immersion rectal ESD workflow rather than a separately developed technique.

Flushing ESD procedures were performed using the Flushing Forced Method during normal saline immersion (Figure 1a). Electrosurgery was delivered using an Erbe VIO3 electrosurgical unit.

The procedures were performed by two endoscopists, one of whom had performed more than 500 ESD procedures and the other approximately 50.

Mucosal incision and submucosal dissection were performed using either high‐cut effect 4.0 or endoCUT I (effect 2; duration 2; interval 10). Pre‐sealing and hemostatic management of visible vessels were performed using Forced Coagulation effect 6.5. Knife‐tip Forced Coagulation was the first‐line approach for the management of active bleeding. Hemostatic forceps were used when bleeding persisted despite knife‐tip coagulation. The decision to switch devices was made at the operator's discretion, without a prespecified threshold. Hemostatic forceps were not used routinely for prophylactic vessel pre‐coagulation.

### 2.4 Outcomes

The principal measure of technical feasibility was successful completion of rectal Flushing ESD with en bloc resection, without abandonment of the procedure or conversion to surgery. Video‐derived treated visible submucosal vessel burden, minor bleeding episodes, red‐out episodes, and hemostatic forceps use were treated as descriptive procedural metrics. Procedure duration, resection area, and resection speed were also reported descriptively.

Minor bleeding (ooze) was defined a priori as persistent low‐flow bleeding requiring further applications of electrosurgical energy with the knife tip. Bleeding episodes causing loss of visualization (red‐out) were defined as rapid hemorrhage causing loss of endoscopic visualization. Visible submucosal vessels were classified according to their estimated diameter relative to the knife dimensions. Vessels with a diameter greater than or equal to the 0.9‐mm knife ball tip were classified as treated visible submucosal vessels when they were first subjected to deliberate electrosurgical intervention. Intervention included pre‐coagulation, cutting, dissection, or any other electrosurgical application. Larger treated vessels were further classified according to whether their diameter was greater than or equal to the 2.7‐mm shaft diameter. Each treated vessel was counted once only, irrespective of repeated electrosurgical applications or its subsequent reappearance during the procedure. Bleeding and hemostatic outcomes were summarized per procedure, per treated visible vessel, and per unit resection area.

Completeness of resection was additionally assessed by R0 resection. R0 resection was defined as en bloc resection with histologically negative horizontal and vertical margins. Curative endoscopic resection was defined as en bloc R0 resection without histopathological high‐risk features indicating a requirement for additional oncological treatment, including deep submucosal invasion, lymphovascular invasion, poorly differentiated histology, or grade 2 or 3 tumor budding. Safety outcomes included intraprocedural perforation, delayed bleeding, blood transfusion, post‐ESD coagulation syndrome, unplanned repeat endoscopy or other intervention related to the procedure, and unplanned hospital readmission. Procedural and post‐procedural safety outcomes were obtained from the institutional electronic medical record.

### 2.5 Data Collection

The ten procedural videos were allocated among three reviewers, who assessed three, three, and four videos, respectively. Each video was assessed by one reviewer only, and no duplicate independent review was undertaken. The reviewers recorded treated visible submucosal vessels, minor bleeding episodes, red‐out events, and the number of vessels treated using hemostatic forceps, classified as therapeutic or prophylactic. Because the videos were not reviewed in duplicate, no disagreement‐resolution procedure was required, and interobserver agreement was not assessed. Reviewers were not blinded, as the technique was apparent from the video.

Additional procedural and lesion data were extracted from the institutional electronic medical record and prospectively maintained ESD database. These included age, sex, lesion location, lesion morphology, Japan NBI Expert Team classification, histological outcome, resection technique, lesion dimensions, resection duration, and resection area.

The resected specimens were measured after pinning onto a cork board. Resection area was estimated using the formula for the area of an ellipse (πLS/4), based on the two reported maximum dimensions (long axis [*L*] and short axis [*S*]). Resection speed was calculated by dividing the ellipse‐estimated resection area by procedure duration (cm^2^/min).

Saline irrigation volumes were not recorded in a standardized manner and could not be analyzed. This is a limitation, as irrigation volume may influence visualization, procedural flow, and hemostatic performance.

### 2.6 Statistical Methods and Reporting

All analyses were descriptive. Categorical variables are reported as counts and percentages. Given the small sample size and right‐skewed distributions, continuous variables are presented primarily as medians with interquartile ranges. Means are also provided for completeness.

## 3 Results

### 3.1 Participants and Lesion Characteristics

Ten rectal Flushing ESD procedures were included. Patient and lesion characteristics are shown in Table 1.

**TABLE 1:** Patient, lesion, procedural, vascular, and hemostatic characteristics of the Flushing ESD cohort. Values are presented as number (%) or median (interquartile range), mean, as appropriate. Resection speed was calculated using the ellipse‐estimated area of the pinned resected specimen rather than directly measured specimen surface area. A visible submucosal vessel was counted when it was first subjected to electrosurgical treatment and was counted once only, irrespective of repeated treatment or subsequent reappearance. Intervention included pre‐coagulation, cutting, dissection, or any other electrosurgical application. Visible vessels that did not undergo electrosurgical treatment were not included. Treated vessels were classified as having a diameter greater than or equal to the 0.9‐mm knife ball tip, with larger treated vessels defined as those with a diameter greater than or equal to the 2.7‐mm shaft diameter. Bleeding‐related events and hemostatic forceps use are reported per procedure and normalized per treated visible vessel and per cm^2^ of resection area. ESD, endoscopic submucosal dissection; JNET, Japan NBI Expert Team; LST‐G MN, laterally spreading tumor, granular mixed nodular type; LST‐NG, laterally spreading tumor, non‐granular type; LGD, low‐grade dysplasia; HGD, high‐grade dysplasia; IQR, interquartile range; R0, histologically complete resection with negative horizontal and vertical margins; Paris 0‐Is, sessile protruding lesion; Paris 0‐Isp, subpedunculated lesion; Paris 0‐IIa+IIc, slightly elevated lesion with a depressed component.

Table 1	Flushing ESD (*n* = 10)
Patient Characteristics	
Male: *N* (%)	7 (70%)
Age: Median (IQR), Mean	72.5 (56–79), 67.6
Lesion Characteristics	
Location:	
High Rectum: *N* (%)	3 (30%)
Mid Rectum: *N* (%)	2 (20%)
Low‐mid Rectum: *N* (%)	3 (30%)
Low Rectum: *N* (%)	2 (20%)
JNET Classification	
JNET 2A: *N* (%)	8 (80%)
JNET 2B: *N* (%)	2 (20%)
Morphology	
LST‐G MN: *N* (%)	6 (60%)
LST‐NG: *N* (%)	1 (10%)
0‐Is: *N* (%)	1 (10%)
0‐Isp: *N* (%)	1 (10%)
0‐IIa+IIc: *N* (%)	1 (10%)
Histology	
LGD: *N* (%)	4 (40%)
HGD: *N* (%)	5 (50%)
Carcinoma: *N* (%)	1 (10%)
Lesion Size (mm): Median (IQR), Mean	55 (40–69.5), 63
Vessel Metrics	
Number of Treated Vessels with Diameter ≥Knife Ball Tip (0.9 mm): Median (IQR), Mean	21 (11.5–40.75), 28.3
Number of Treated Vessels per cm^2^: Median (IQR), Mean	1.04 (0.64–1.74), 1.38
Number of Treated Vessels with Diameter ≥Knife Ball Tip (0.9 mm) but < Shaft Diameter (2.7 mm): Median (IQR), Mean	21 (11.5–39.25), 27.6
Number of Treated Vessels with Diameter ≥ Shaft Diameter (2.7 mm): Median (IQR), Mean	0 (0–0.75), 0.7
Procedural Characteristics	
Resection Area (cm^2^): Median (IQR), Mean	20 (13–29), 38.2
Procedure Duration (minutes): Median (IQR), Mean	102.5 (100–167.5), 130.5
Speed of Resection (cm^2^/min): Median (IQR), Mean	0.14 (0.13–0.25), 0.23
Completeness of Resection and Safety Outcomes	
En Bloc Resection: *N* (%)	10 (100%)
R0 Resection: *N* (%)	10 (100%)
Curative Endoscopic Resection: *N* (%)	9 (90%)
Intraprocedural Perforation: *N* (%)	0 (0%)
Delayed Bleeding: *N* (%)	0 (0%)
Post‐ESD Coagulation Syndrome: *N* (%)	0 (0%)
Unplanned Repeat Endoscopy or Other Intervention: *N* (%)	0 (0%)
Blood Transfusion: *N* (%)	0 (0%)
Unplanned Hospital Readmission: *N* (%)	0 (0%)
Hemostasis and Bleeding Outcomes	
Hemostatic Forceps Use (Therapeutic): Median (IQR), Mean	0 (0–0), 0.1
Hemostatic Forceps Use (Therapeutic) per Treated Visible Vessel: Median (IQR), Mean	0 (0–0), 0.001
Hemostatic Forceps Use (Therapeutic) per cm^2^: Median (IQR), Mean	0 (0–0), 0.001
Hemostatic Forceps Use (Prophylactic): Median (IQR), Mean	0 (0–0), 0.1
Hemostatic Forceps Use (Prophylactic) per Treated Visible Vessel: Median (IQR), Mean	0 (0–0), 0.001
Hemostatic Forceps Use (Prophylactic) per cm^2^: Median (IQR), Mean	0 (0–0), 0.001
Minor Bleeding Episodes (ooze): Median (IQR), Mean	17 (10–29.8), 20.4
Ooze per Treated Visible Vessel: Median (IQR), Mean	0.7 (0.6–1), 0.8
Ooze per cm^2^: Median (IQR), Mean	0.8 (0.6–1.2), 1.2
Bleeding Episodes Causing Transient Loss of Visualization (Red‐out): Median (IQR), Mean	4 (3–6.8), 5
Red‐out per Treated Visible Vessel: Median (IQR), Mean	0.15 (0.11–0.26), 0.24
Red‐out per cm^2^: Median (IQR), Mean	0.2 (0.11–0.23), 0.38

### 3.2 Procedural Characteristics

The median procedure duration was 102.5 min (interquartile range [IQR] 100–167.5; mean 130.5 min). The median resection speed was 0.14 cm^2^/min (IQR 0.13–0.25; mean 0.23 cm^2^/min). Other procedural characteristics are shown in Table 1.

### 3.3 Completeness of Resection and Safety Outcomes

En bloc and R0 resection were achieved in all 10 cases (100%), while curative endoscopic resection was achieved in nine cases (90%). The single non‐curative resection was an R0‐resected carcinoma with a submucosal invasion depth of 1800 µm, without lymphovascular invasion or tumor budding. The patient subsequently received radiotherapy for rectal cancer, coordinated with planned radiotherapy for concomitant prostate cancer. There were no intraprocedural perforations, delayed bleeding events, blood transfusions, cases of post‐ESD coagulation syndrome, unplanned repeat endoscopic or other interventions related to the procedure, or unplanned hospital readmissions.

### 3.4 Vessel Metrics

The median number of treated visible vessels with diameter greater than or equal to the knife ball tip was 21 per case (IQR 11.5–40.75; mean 28.3). This corresponded to a median of 1.04 vessels/cm^2^ (IQR 0.64–1.74; mean 1.38 vessels/cm^2^). Other vascular metrics are shown in Table 1.

### 3.5 Hemostatic Forceps Utilization

Hemostatic forceps were used therapeutically for active bleeding from one vessel in one procedure, after bleeding persisted despite knife‐tip coagulation. During the same procedure, the forceps were also used prophylactically to pre‐coagulate one further vessel because the device had already been deployed and remained in the working channel. No prophylactic hemostatic forceps use occurred in the other nine procedures.

### 3.6 Bleeding Outcomes

Minor bleeding episodes were observed during Flushing ESD and were managed endoscopically using further applications of electrosurgical energy with the knife tip. The median number of minor bleeding episodes was 17 per case (IQR 10–29.8; mean 20.4). The median number of minor bleeding episodes per treated visible vessel was 0.7 (IQR 0.6–1.0; mean 0.8), and the median number per cm^2^ was 0.8 (IQR 0.6–1.2; mean 1.2).

Bleeding episodes causing transient loss of visualization were also recorded. The median number of red‐out events was 4 per case (IQR 3–6.8; mean 5). The median number of red‐out events per treated visible vessel was 0.15 (IQR 0.11–0.26; mean 0.24), and the median number per cm^2^ was 0.20 (IQR 0.11–0.23; mean 0.38).

All bleeding events were managed endoscopically. No case required abandonment of the procedure or conversion to surgery. Case‐level demographic, lesion, procedural, vascular, and hemostatic data are presented in Table S1.

## 4 Discussion

### 4.1 Principal Findings and Added Contribution

This retrospective video‐based series describes Flushing ESD at a European tertiary referral center. The technique was feasible in all included cases, with en bloc and R0 resection in all 10 procedures, while enabling systematic assessment of treated visible vessels, bleeding, red‐out episodes, and hemostatic instrument use.

### 4.2 Interpretation of Bleeding and Hemostatic Outcomes

Minor bleeding and red‐out episodes occurred but were controlled endoscopically. Hemostatic forceps were required therapeutically for one vessel. Without a comparator, neither event frequency nor forceps use can be interpreted as favorable or unfavorable relative to other ESD approaches. Comparison is further limited by the absence of standardized definitions for intraprocedural bleeding in colorectal ESD, where bleeding controlled by knife‐tip coagulation is often not considered a complication.

Published hemostatic metrics provide limited context. Ishikawa et al., comparing Swift and Spray coagulation, reported median intraoperative bleeding counts of 6 (IQR 2–8) and 5 (IQR 3–6) per gastric ESD lesion, with forceps required in 32.7% and 13.8% of events, respectively [4]. In a randomized colorectal study, Nagata et al. reported no forceps use in 68.1% of conventional and 57.1% of underwater ESD procedures [5].

These benchmarks are not directly comparable because of differences in anatomical site, lesion characteristics, definitions, electrosurgical strategy, and video‐counting methods. Although therapeutic forceps use occurred in only one of 10 procedures, Nagata et al. reported one or more hemostatic forceps interventions in 31.9% of conventional and 42.9% of underwater ESD procedures [5]. Comparable denominators for treated visible‐vessel density, bleeding episodes per treated vessel, and red‐out events are unavailable. The values reported here should therefore be regarded as exploratory metrics rather than comparative benchmarks.

In colorectal ESD, Kanazawa et al. reported reduced hemostatic forceps utilization following the introduction of spray coagulation for intraprocedural hemostasis [6]. This finding illustrates that hemostatic forceps use may be influenced by electrosurgical strategy as well as by the immersion environment, operator expertise, lesion characteristics, and thresholds for device exchange.

### 4.3 Potential Mechanisms

The proposed rationale for Flushing ESD is that synchronized saline flushing from the knife tip may disperse micro‐bubbles that accumulate during electrosurgical energy application in a saline environment, potentially reducing spark discharge and facilitating more controlled energy delivery to the target tissue. The limited use of hemostatic forceps observed in this series could reflect the saline immersion environment, improved visualization associated with flushing, the hypothesized reduction in micro‐bubble accumulation and spark discharge, operator expertise, individual thresholds for switching to hemostatic forceps, lesion characteristics, case selection, or a combination of these factors. If Flushing ESD contributed to the observed findings, improved visualization and the proposed effect on micro‐bubble accumulation and energy delivery may both have played a role. Their relative contributions, and any effect of Flushing ESD itself, cannot be determined from the present study and remain hypothetical. Figure 1 illustrates the proposed mechanism.

### 4.4 Strengths and Limitations

A strength of this series is the systematic video‐derived assessment of treated visible submucosal vessel burden, bleeding events, red‐out episodes, and hemostatic instrument utilization. Normalizing these measures to treated visible‐vessel burden and resection area provides a quantitative descriptive framework that may inform the selection of candidate endpoints for future prospective studies, because bleeding burden during ESD is likely to depend on both lesion size and vascular density.

This study has important limitations. First, the sample size was small, and the study was retrospective. Second, inclusion was restricted to 10 of the 13 Flushing ESD procedures (76.9%) for which complete procedural video was available. Detailed reasons for the absence or incompleteness of the remaining three recordings were unavailable, and the included procedures may therefore not be representative of all Flushing ESD procedures performed during the study period. If recordings were more likely to be retained for procedures that proceeded smoothly, the observed frequency or severity of bleeding‐related events may have been underestimated, although the direction of any selection bias cannot be determined. Third, each procedural video was assessed by only one of three reviewers, with no duplicate independent review or formal assessment of interobserver agreement. Reviewer‐related variability in the identification of treated vessels and the classification of minor bleeding and red‐out events therefore cannot be estimated. In addition, the vessel count included only visible vessels that underwent electrosurgical treatment and should not be interpreted as a measure of total visible submucosal vascular burden or vascular density. Reviewers were not blinded, and some authors were involved in the development of the technique. Because classification of minor bleeding and red‐out events involves subjective judgment, knowledge of the technique and investigator involvement may have influenced the recorded event frequencies. Fourth, important bleeding risk factors, including comorbidity, coagulation parameters, fibrosis, and detailed intra‐procedural irrigation volume, were not systematically captured. Fifth, the study was restricted to rectal ESD. Scope stability, access, and adverse event profiles differ in the colon, particularly in the right colon, and the findings should not be generalized beyond rectal ESD. Sixth, this study was descriptive and did not include a valid comparator group; therefore, it cannot support conclusions regarding comparative efficacy, safety, resection speed, or hemostatic superiority. Seventh, although no intraprocedural perforation, delayed bleeding, post‐ESD coagulation syndrome, or unplanned repeat intervention was observed, the small sample size precludes conclusions regarding the safety of Flushing ESD or the incidence of uncommon adverse events. Finally, the absence of standardized published comparators for minor oozing, red‐out events, and treated visible‐vessel density limits the external interpretation of these exploratory video‐derived endpoints.

### 4.5 Future Research

Future studies should prospectively compare Flushing ESD with standard ESD using predefined criteria for hemostatic forceps use and bleeding‐related visual interruption, standardized irrigation‐volume capture, relevant bleeding‐risk variables, duplicate independent video assessment by reviewers uninvolved in development or performance of the technique, predefined vessel‐tracking criteria, consensus or third‐reviewer adjudication, and formal assessment of interobserver agreement. The video‐derived measures of treated visible‐vessel burden, minor bleeding, red‐out, and hemostatic forceps use reported here may serve as candidate endpoints.

## 5 Conclusion

In this small retrospective series, rectal Flushing ESD was technically feasible, with en bloc resection achieved in all 10 cases and all bleeding‐related events controlled endoscopically. These descriptive findings do not establish comparative hemostatic benefit and provide feasibility data to inform future prospective comparative studies.

## Author Contributions

NS, HB, and TT contributed to the conceptualization of the study. TM, JL, and NS were responsible for data curation, while TM performed the formal analysis. Investigation was undertaken by TM, JL, and NS, and the methodology was developed by TM, JL, and NS. Project administration was conducted by TM, JL, and NS, and resources were provided by JL and NS. TM created the visualizations. NS and TT provided supervision throughout the project. TM drafted the original manuscript, and all authors critically reviewed and edited it, approved the final version, and agreed to be accountable for all aspects of the work.

## Funding

This study received no specific funding. Imperial College London will pay the open access article processing charge.

## Ethics Statement

**Approval of the research protocol by an Institutional Review Board**: N/A. This project was approved as a local service evaluation by the Quality Improvement Committee of London North West University Healthcare NHS Trust (reference: R1K‐CCON‐2025‐0018). NHS Research Ethics Committee review was not required.

## Consent

Written informed consent was obtained from all patients prior to their procedure to allow the use of anonymized data for research and publication.

## Conflicts of Interest

Thomas Matthews and Hiromitsu Ban declare no conflicts of interest. Jonathan Landy has received research funding from Perspectum Limited and Odin Vision. Takashi Toyonaga has received research funding from Fujifilm Corporation, Kaneka Corporation, Top Corporation and Olympus Corporation, and has received patent royalties from Olympus Corporation and Fujifilm Corporation. Noriko Suzuki has received faculty fees from Olympus Corporation and Fujinon.

## Supporting information




**TABLE S1A–D** Case‐level patient, lesion, procedural, vascular, bleeding, and hemostatic outcomes for the 10 analyzed Flushing ESD procedures.

**VIDEO S1** Flushing‐assisted saline immersion endoscopic submucosal dissection. Saline immersion provides buoyancy and improves visualization of the submucosal plane. During electrosurgical energy application, saline is flushed synchronously from the knife tip (FlushKnife BTs, Fujifilm), a maneuver hypothesized to disperse micro‐bubbles, reduce electrical spark discharge, and facilitate controlled dissection and targeted hemostasis.
**VIDEO S1** alt text
Endoscopic video of flushing‐assisted saline immersion endoscopic submucosal dissection in the rectum. Saline immersion maintains buoyancy and visualization of the submucosal plane. During electrosurgical energy application, saline is flushed synchronously from the FlushKnife BTs tip, a maneuver hypothesized to disperse micro‐bubbles, reduce spark discharge, and facilitate controlled dissection and targeted hemostasis.

## Data Availability

De‐identified data and annotated video‐derived metrics can be made available from the corresponding author on reasonable request.
