# Supplementary material for: Flushing‐Assisted Saline Immersion Endoscopic Submucosal Dissection in the Rectum: A Video‐Based Feasibility Series
Source: DEN Open. 2026 Sep 30;7(1):e70447. doi: 10.1002/deo2.70447 (PMC13627967; doi:10.1002/deo2.70447)
Supplement: Supplementary file 1 [file DEO2-7-e70447-s001.docx]

| Supplementary Table S1A | Flushing ESD (n=10) | | | | | | | | | |
| --- | --- | --- | --- | --- | --- | --- | --- | --- | --- | --- |
|  | | | | | | | | | | |
| Patient and lesion characteristics | | | | | | | | | | |
| Characteristic | Case 1 | Case 2 | Case 3 | Case 4 | Case 5 | Case 6 | Case 7 | Case 8 | Case 9 | Case 10 |
| Age, years | 84 | 52 | 50 | 69 | 83 | 75 | 75 | 38 | 70 | 80 |
| Sex | Female | Female | Male | Male | Male | Male | Male | Female | Male | Male |
| Location | Low to mid rectum | High rectum | High rectum | Low to mid rectum | Low to mid rectum | Low rectum | Low rectum | Mid rectum | Mid rectum | High rectum |
| Morphology | LST-G MN | LST-G MN | LST-NG | 0-Is | LST-G MN | 0-IIa+IIc | LST-G MN | 0-Isp | LST-G MN | LST-G MN |
| JNET classification | Type 2B | Type 2A | Type 2A | Type 2A | Type 2A | Type 2B | Type 2A | Type 2A | Type 2A | Type 2A |
| Lesion size, mm | 60 | 70 | 68 | 50 | 170 | 12 | 40 | 40 | 100 | 20 |
| Histology | TVA with HGD | TVA with HGD | TA with LGD | TVA with HGD | TVA with HGD | Carcinoma | TVA with LGD | TVA with HGD | TA with LGD | TVA with LGD |
|  | | | | | | | | | | |
| Supplementary Table S1A. Case-level patient and lesion characteristics. HGD, high-grade dysplasia; JNET, Japan NBI Expert Team; LGD, low-grade dysplasia; LST-G MN, laterally spreading tumor, granular mixed nodular type; LST-NG, laterally spreading tumor, non-granular type; TA, tubular adenoma; TVA, tubulovillous adenoma; Paris 0-Is, sessile protruding lesion; Paris 0-Isp, subpedunculated lesion; Paris 0-IIa+IIc, slightly elevated lesion with a depressed component. | | | | | | | | | | |

| Supplementary Table S1B | Flushing ESD (n=10) | | | | | | | | | |
| --- | --- | --- | --- | --- | --- | --- | --- | --- | --- | --- |
|  | | | | | | | | | | |
| Procedural and resection outcomes | | | | | | | | | | |
| Characteristic | Case 1 | Case 2 | Case 3 | Case 4 | Case 5 | Case 6 | Case 7 | Case 8 | Case 9 | Case 10 |
| Pinned specimen dimensions, mm | 60 × 55 | 70 × 50 | 75 × 50 | 50 × 32 | 170 × 130 | 25 × 15 | 45 × 40 | 40 × 45 | 110 × 90 | 25 × 20 |
| Ellipse-estimated resection area, cm² | 25.92 | 27.49 | 29.45 | 12.57 | 173.57 | 2.95 | 14.14 | 14.14 | 77.75 | 3.93 |
| Procedure duration, min | 100 | 200 | 130 | 100 | 240 | 90 | 100 | 105 | 180 | 60 |
| Resection speed, cm²/min | 0.259 | 0.137 | 0.227 | 0.126 | 0.723 | 0.033 | 0.141 | 0.135 | 0.432 | 0.066 |
| En bloc resection | Yes | Yes | Yes | Yes | Yes | Yes | Yes | Yes | Yes | Yes |
| R0 resection | Yes | Yes | Yes | Yes | Yes | Yes | Yes | Yes | Yes | Yes |
| Curative endoscopic resection | Yes | Yes | Yes | Yes | Yes | No | Yes | Yes | Yes | Yes |
| Procedural or post-procedural adverse events | None | None | None | None | None | None | None | None | None | None |
|  | | | | | | | | | | |
| Supplementary Table S1B. Case-level procedural and resection outcomes. Resection area was estimated using the formula for an ellipse, π × (long axis/2) × (short axis/2), based on the two maximum dimensions of the pinned specimen. Resection speed was calculated as ellipse-estimated resection area divided by procedure duration. R0, histologically complete en bloc resection with negative horizontal and vertical margins. Case 6 had an R0 resection but was classified as having undergone non-curative endoscopic resection because the carcinoma had submucosal invasion to 1,800 µm. Lymphovascular invasion and tumor budding were absent. The patient subsequently received radiotherapy for rectal cancer, coordinated with planned radiotherapy for concomitant prostate cancer. Adverse events assessed were intraprocedural perforation, delayed bleeding, blood transfusion, post-ESD coagulation syndrome, unplanned repeat endoscopy or other intervention related to the procedure, and unplanned hospital readmission. | | | | | | | | | | |

| Supplementary Table S1C | Flushing ESD (n=10) | | | | | | | | | |
| --- | --- | --- | --- | --- | --- | --- | --- | --- | --- | --- |
|  | | | | | | | | | | |
| Treated visible-vessel and bleeding outcomes | | | | | | | | | | |
| Characteristic | Case 1 | Case 2 | Case 3 | Case 4 | Case 5 | Case 6 | Case 7 | Case 8 | Case 9 | Case 10 |
| Treated vessels ≥ 0.9 and < 2.7 mm, n | 40 | 26 | 53 | 11 | 65 | 8 | 7 | 16 | 37 | 13 |
| Treated vessels ≥2.7 mm, n | 0 | 0 | 0 | 0 | 2 | 0 | 1 | 0 | 4 | 0 |
| Total treated visible vessels, n | 40 | 26 | 53 | 11 | 67 | 8 | 8 | 16 | 41 | 13 |
| Treated visible vessels/cm² | 1.543 | 0.946 | 1.800 | 0.875 | 0.386 | 2.712 | 0.566 | 1.132 | 0.527 | 3.308 |
| Minor bleeding episodes, n | 20 | 30 | 38 | 10 | 40 | 14 | 8 | 10 | 29 | 5 |
| Minor bleeding/visible vessel | 0.500 | 1.154 | 0.717 | 0.909 | 0.597 | 1.750 | 1.000 | 0.625 | 0.707 | 0.385 |
| Minor bleeding/cm² | 0.772 | 1.091 | 1.290 | 0.796 | 0.230 | 4.746 | 0.566 | 0.707 | 0.373 | 1.272 |
| Red-out events, n | 5 | 3 | 6 | 3 | 12 | 7 | 3 | 1 | 9 | 1 |
| Red-out/treated visible vessel | 0.125 | 0.115 | 0.113 | 0.273 | 0.179 | 0.875 | 0.375 | 0.062 | 0.220 | 0.077 |
| Red-out/cm² | 0.193 | 0.109 | 0.204 | 0.239 | 0.069 | 2.373 | 0.212 | 0.071 | 0.116 | 0.254 |
|  | | | | | | | | | | |
| Supplementary Table S1C. Case-level treated visible-vessel and bleeding outcomes. Visible submucosal vessels were defined as treated vessels with a diameter greater than or equal to the 0.9 mm knife ball tip. Larger vessels were defined as those with a diameter greater than or equal to the 2.7 mm shaft. Each vessel was counted once, at the time of its first electrosurgical treatment. Minor bleeding was defined as persistent low-flow bleeding requiring further electrosurgical applications with the knife tip. Red-out was defined as rapid bleeding causing transient loss of endoscopic visualization. Area-normalized values use the ellipse-estimated resection area. | | | | | | | | | | |

| Supplementary Table S1D | Flushing ESD (n=10) | | | | | | | | | |
| --- | --- | --- | --- | --- | --- | --- | --- | --- | --- | --- |
|  | | | | | | | | | | |
| Therapeutic and prophylactic hemostatic forceps use | | | | | | | | | | |
| Characteristic | Case 1 | Case 2 | Case 3 | Case 4 | Case 5 | Case 6 | Case 7 | Case 8 | Case 9 | Case 10 |
| Therapeutically treated vessels, n | 0 | 0 | 0 | 0 | 1 | 0 | 0 | 0 | 0 | 0 |
| Therapeutic use/treated visible vessel | 0.000 | 0.000 | 0.000 | 0.000 | 0.015 | 0.000 | 0.000 | 0.000 | 0.000 | 0.000 |
| Therapeutic use/cm² | 0.000 | 0.000 | 0.000 | 0.000 | 0.006 | 0.000 | 0.000 | 0.000 | 0.000 | 0.000 |
| Prophylactically treated vessels, n | 0 | 0 | 0 | 0 | 1 | 0 | 0 | 0 | 0 | 0 |
| Prophylactic use/treated visible vessel | 0.000 | 0.000 | 0.000 | 0.000 | 0.015 | 0.000 | 0.000 | 0.000 | 0.000 | 0.000 |
| Prophylactic use/cm² | 0.000 | 0.000 | 0.000 | 0.000 | 0.006 | 0.000 | 0.000 | 0.000 | 0.000 | 0.000 |
|  | | | | | | | | | | |
| Supplementary Table S1D. Case-level therapeutic and prophylactic hemostatic forceps use. Hemostatic forceps use represents the number of vessels treated, not the number of electrosurgical activations. Knife-tip Forced Coagulation was the first-line approach for active bleeding. In case 5, hemostatic forceps were used therapeutically for one vessel when bleeding persisted despite knife-tip coagulation and prophylactically for one further vessel because the forceps had already been deployed and remained in the working channel. No other procedure involved hemostatic forceps use. ESD, endoscopic submucosal dissection. | | | | | | | | | | |
